# Graphical Optimization of Spectral Shift Reconstructions for Optical Backscatter Reflectometry

**DOI:** 10.3390/s21186154

**Published:** 2021-09-14

**Authors:** Daniel C. Sweeney, Dennis M. Sweeney, Christian M. Petrie

**Affiliations:** 1 Oak Ridge National Laboratory, Oak Ridge, TN 37831, USA; petriecm@ornl.gov; 2 Department of Mathematics, The Ohio State University, Columbus, OH 42310, USA; sweeney.427@osu.edu

**Keywords:** optical backscatter reflectometry, optical frequency domain reflectometry, optical fibers, distributed sensing, graph signal processing, adaptive methods

## Abstract

Optical backscatter reflectometry (OBR) is an interferometric technique that can be used to measure local changes in temperature and mechanical strain based on spectral analyses of backscattered light from a singlemode optical fiber. The technique uses Fourier analyses to resolve spectra resulting from reflections occurring over a discrete region along the fiber. These spectra are cross-correlated with reference spectra to calculate the relative spectral shifts between measurements. The maximum of the cross-correlated spectra—termed *quality*—is a metric that quantifies the degree of correlation between the two measurements. Recently, this quality metric was incorporated into an adaptive algorithm to (1) selectively vary the reference measurement until the quality exceeds a predefined threshold and (2) calculate incremental spectral shifts that can be summed to determine the spectral shift relative to the initial reference. Using a graphical (network) framework, this effort demonstrated the optimal reconstruction of distributed OBR measurements for all sensing locations using a maximum spanning tree (MST). By allowing the reference to vary as a function of both time and sensing location, the MST and other adaptive algorithms could resolve spectral shifts at some locations, even if others can no longer be resolved.

## 1. Introduction

Recent advances in sensing technologies and signal processing have enabled spatially distributed measurements that could greatly increase capabilities in environmental condition monitoring across a wide range of science and engineering disciplines. In addition to their ability to perform spatially distributed measurements, fiber optic sensors also offer a small profile, immunity to electromagnetic interference, and the capability for remote operation. Many different interrogation techniques have been demonstrated for distributed fiber optic sensing, most of which use either optical time domain reflectometry (OTDR) or optical frequency domain reflectometry (OFDR). Fiber optic interrogation techniques are further delineated based on the scattering mechanism (e.g., Rayleigh, Raman, and Brillouin scattering) by which injected light is reflected back to the photodetectors [1].

OFDR is attractive for applications that require high spatial resolution (on the order of millimeters to centimeters) over distances on the order of meters to tens of meters. For example, OFDR-based distributed sensing has been explored for monitoring strain during welding [2] and solidification [3]; temperature distributions for cryogenic applications [4] and inside catalytic reactors [5], flowing coolant loops [6,7,8], and energized transformer cores [9]; local power deposition in nuclear reactors [10,11]; and liquid level [12,13]. More recently, fiber optic sensors have been embedded into metals [14,15,16,17] and ceramics [18,19] using additive manufacturing technologies to directly monitor spatially distributed strain in harsh environments, such as those found in nuclear reactors, where fiber could be exposed to high temperatures, radiation, or chemically aggressive media.

The random density fluctuations, or scattering centers, created along an optical fiber’s length during its fabrication can be interrogated using a tunable laser that is swept over a range of frequencies. Each scattering center produces a low-amplitude reflection that can be used in aggregate to produce a scattering profile unique to each fiber. This profile can be resolved along the length of the fiber and varies with applied temperature and strain, thus enabling the use of optical fibers as distributed sensors. Because the shift in an optical fiber sensor’s backscatter profile is indicative of a change in temperature or strain, OFDR measurements are performed by comparing the backscatter profile from a fiber under test (FUT) with the backscatter profile from the same fiber under known conditions and calculating the shift in the optical frequency spectrum between the two measurements [20].

Conventionally, backscatter profiles recorded from an FUT as a function of time are compared with a single reference profile to reconstruct the spectral shifts as a function of time and position. However, because the Rayleigh backscatter signal is relatively weak (compared to Bragg gratings, for example), the low SNR makes it difficult to reliably determine large spectral shifts that result from large changes in temperature or strain. Using a static reference, previous works have resolved steady-state changes in temperature and strain in unaltered singlemode fiber of approximately 650 °C [21] and $20\times 10^{3}$ microstrain [15], respectively. Transient measurements have been made at significantly higher temperatures (∼2100 °C), but only for times on the order of seconds [22]. Heinze et al. demonstrated that using a static reference measurement was not a viable approach to measuring the shrinkage in hardening epoxy [3]. Physically inscribing features, such as fiber Bragg gratings (FBGs), has enabled temperature measurements up to 1100 °C, at the cost of lower spatial resolution [23]. Machine learning techniques, such as the use of artificial neural networks, have been developed for backscatter-based distributed optical fiber sensors to improve feature recognition toward vibration monitoring [24,25,26]. Genetic algorithms have also been explored to measure hoop strain using distributed optical fiber sensors in pipelines to detect leaks [27].

Other signal processing methods have also been explored to improve OFDR analysis. Cheng et al. and Luo et al. both demonstrated strategies for compensating for the cumulative effects of expansion and thermo-optic effects along the length of the fiber [28,29]. Fan et al. compensated for phase noise in OFDR measurements using measurements from an auxiliary interferometer in addition to those from the FUT [30]. Heinze et al. and Chen et al. were able to increase the range of resolvable spectral shifts (strain in this case) by setting the reference to the measurement immediately preceding each new measurement and summing incremental spectral shifts across all times [3,22]. Bado et al. and Sweeney et al. recently demonstrated an adaptive scheme to dynamically vary the reference measurement based on the degree of correlation, or quality, between the target and reference measurements [31,32]. Sweeney’s adaptive scheme, termed the inchworm algorithm, was successful in resolving temperatures during furnace testing up to approximately 1000 °C and during five days of irradiation inside a nuclear test reactor.

Building on the concept of adaptive methods based on correlation metrics, this work focuses on the application of OFDR-based distributed sensing in harsh environments, with an emphasis on improving data processing and reconstruction from existing distributed fiber optic sensors. A deterministic method to provably optimize the spectral shift reconstruction of a distributed OFDR-based optical fiber sensor is demonstrated using a maximum spanning tree to select the best reference measurements for each active measurement in a series. This method is applied to distributed optical fiber sensors with and without FBGs and is capable of resolving spectral shifts of up to approximately −2000 GHz at 1130 °C and −1600 GHz at 950 °C, respectively. The results presented in this work provide a method to calculate an optimal spectral shift reconstruction for OFDR measurements which can be used alone or as a comparison standard for future analysis techniques.

## 2. Background

### 2.1. Measurement Theory

**Figure 1:**
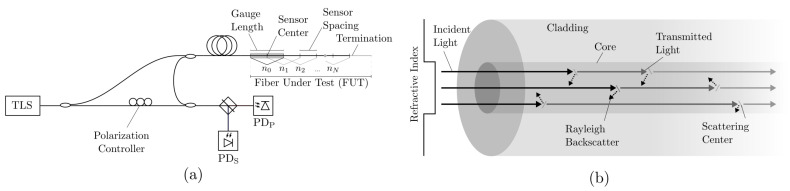
(**a**) Optical backscatter reflectometry (OBR) schematic showing how a tunable laser source (TLS) is used to produce an interference pattern resulting from Rayleigh backscatter along the fiber under test (FUT). The *S*- and *P*-polarization states are recorded separately by photodiodes $PD_{S}$ and $PD_{P}$, respectively. The Fourier-transformed measurements are discretized into individual *sensors* or *gauges* (with indices $n_{0}$, $n_{1}$, *…*$n_{N}$) that can be analyzed separately to provide spatially distributed measurements. (**b**) Schematic of Rayleigh backscatter in a step-index singlemode optical fiber with light propagating through the fiber and reflected by local scattering centers.

OFDR uses a tunable laser source (TLS) coupled to an interferometer comprising a reference arm and a sensing arm containing the FUT [20]. Reflections occurring along the FUT are caused by Rayleigh backscattering, Fresnel reflections, or Bragg gratings. These reflections interfere with light from the reference arm and are recorded using a photodetector as a function of the optical frequency (ν) of the TLS. The beat frequencies of the recorded spectra are proportional to the location of the backscattered light and can be determined using a Fourier transform (i.e., from the ν domain to the time of flight, or τ, domain). Optical backscatter reflectometry (OBR), a subset of OFDR techniques, maintains the polarization state of the reference fiber and independently measures the two orthogonal polarization states at the output of the interferometer [33]. This polarization-diverse implementation mitigates signal fading caused by polarization misalignment of the interfering measurement and reference fields. Figure 1 shows a schematic of an OBR optical network and the Rayleigh backscatter process.

**Figure 2:**
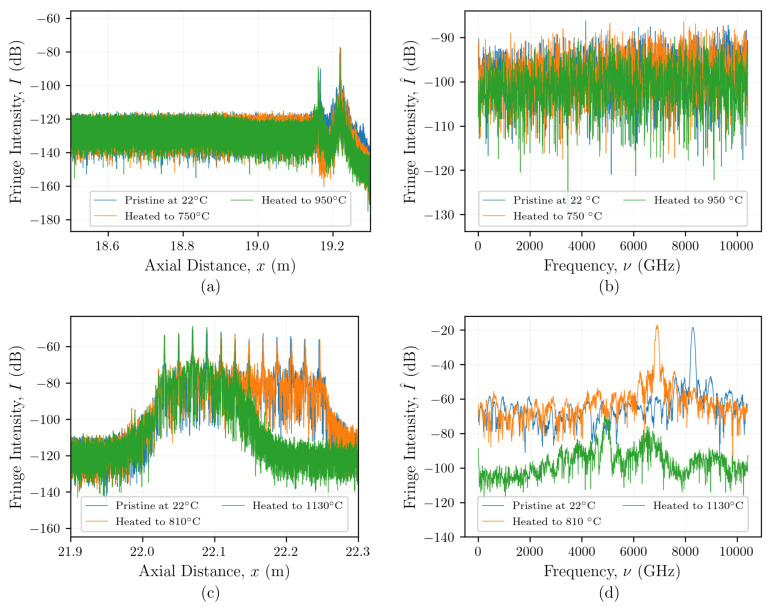
Examples of OBR measurements made during thermal testing using fibers with and without FBGs: (**a**) *x* domain data from a fiber without FBGs at room temperature and after heating to 750 and 950 °C, (**b**) ν domain data from *A* between 19.050 m and 19.060 m, (**c**) *x* domain data from a fiber with inscribed femtosecond Type-II FBGs at room temperature and after heating to 810 and 1130 °C, and (**d**) ν domain data from (**c**) between 22.187 and 22.193 m.

Figure 2a,c show examples of OBR measurements made using Rayleigh backscatter from singlemode fiber without and with inscribed FBGs, respectively. These data are shown after a Fourier transform and conversion to the *x* domain both near room temperature (time t=0 min) and at later times at high temperatures of 950 °C in Figure 2a and 1130 °C in Figure 2c. The high-temperature data presented in Figure 2 are described in greater detail in Section 3. Performing distributed temperature or strain measurements requires windowing of the Fourier transformed OBR spectra (in the τ domain) into *N* discrete *sensors*, each having an index *n*, center position $x_{n}$, and *gauge length* 2Δx (Figure 1). The sensor locations (*x*) are determined from the τ values using the relation $x=c\tau /\left(2\eta_{g}\right)-x_{0},$ where *c* is the speed of light in a vacuum, $\eta_{g}$ is the group index, and $x_{0}$ is the internal path length difference between the two arms of the interferometer (i.e., absent an FUT). This gives intensity measurements $I(x_{n})$ spanning $x\in [x_{n}-\Delta x,x_{n}+\Delta x]$.

The windowed τ domain data from each sensor are then transformed back into the ν-domain to give the spectra resulting only from reflections occurring within a given sensor:

(1) $${\hat{I}}_{n}\left(\nu \right)=F^{-1}\left\{I(x_{n})\right\},$$

where $F^{-1}\left\{\cdot \right\}$ is the inverse Fourier transform operator. Figure 2a,c show examples of spectra (in the ν domain) resulting from reflections only at a single sensor location obtained by transforming one set of the windowed data shown in Figure 2a,c, respectively. This process is iterated across each of the N sensors in the FUT. Under static environmental conditions, the spectra at a given time *t* appear random but are invariant in time. Froggatt shows how this property can be modeled as a weak FBG with a grating amplitude and phase that varies randomly along its length [20]. This model allows ${\hat{I}}_{n}\left(\nu \right)$ to serve as a fingerprint that is unique to a given combination of FUT and optical interrogation system (due to the dependence on launch conditions). Similar to an FBG, changes in temperature or strain affect the “grating” period and group index, resulting in a shift in the spectral content (ν-domain) for each sensor. Therefore, with proper calibration, local spectral shifts can be used to determine changes in temperature and mechanical strain along the fiber’s length. Since Froggatt’s initial work, spatially distributed fiber optic sensing systems based on OBR have now become commercially available and are being used across a wide range of applications. More detailed summaries of OBR-based distributed sensing can be found in the literature [34,35].

### 2.2. Spectral Shift and Quality

OBR-based distributed sensing is used to determine the relative change from one measurement (hereafter referred to as the *target measurement*) relative to some reference scan. For a set of time series measurements $\left\{I\left(t,x_{n}\right):\left(0\leq t\leq T\right)\times \left(0\leq n\leq N\right)\right\}$, the spectral shift of sensor *n* between two measurements taken at times $t_{a}$ and $t_{r}$ such that $t_{r}<t_{a}$, can be calculated as

(2) $$\Delta \nu \left(t_{a},t_{r},x_{n}\right)=\underset{\nu }{\mathrm{argmax}}\left\{{\hat{I}}_{n}\left(t_{a},\nu \right)⊗{\hat{I}}_{n}\left(t_{r},\nu \right)\right\},$$

where Δν is the spectral shift, and ⊗ is the cross-correlation operator [20,31]. The prominence of the cross-correlation peak in Equation (2), which is used to determine Δν, directly affects the reliability of the measurement. If the signal-to-noise ratio (SNR) becomes too low, then the cross-correlation could falsely identify an artificial peak that is unrelated to the spectral shift caused by a thermo-mechanical stimulus. To correct for broadband changes in the reflected light intensity, normalized spectra (${\hat{I}}_{n}^{\prime }\left(t,\nu \right)$) are first determined:

(3) $$\begin{array}{c} {\hat{I}}_{n}^{\prime }\left(t,\nu \right)={\hat{I}}_{n}\left(t,\nu \right)-{\bar{\hat{I}}}_{n}\left(t,\nu \right), \end{array}$$

where ${\bar{\hat{I}}}_{n}\left(t,\nu \right)$ is the frequency-averaged intensity of ${\hat{I}}_{n}\left(t,\nu \right)$ for sensor *n* at time *t*. A metric—referred to as the *spectral shift quality*—quantifies the prominence of the cross-correlation spectrum to characterize the reliability of the spectral shift measurement. The spectral shift quality is given by

(4) $$\begin{array}{c} Q_{n}\left(t_{a},t_{r}\right)=\frac{\max\left\{{\hat{I}}_{n}^{\prime }\left(t_{a},\nu \right)⊗{\hat{I}}_{n}^{\prime }\left(t_{r},\nu \right)\right\}}{∥{\hat{I}}_{n}^{\prime }\left(t_{r},\nu \right){∥}^{2}}, \end{array}$$

where ∥·∥ indicates the $L^{2}$ norm, and $∥{\hat{I}}_{n}^{\prime }\left(t_{r},\nu \right){∥}^{2}$ normalizes the quality relative to the autocorrelation of the reference spectrum, or equivalently, its variance. Until recently, quality was only analyzed by comparing to a heuristic threshold of 0.15 to determine whether or not the calculated spectral shift was reliable [31]. Equation (4) is appropriate when ${\hat{I}}_{n}^{\prime }\left(t_{a},\nu \right)$ and ${\hat{I}}_{n}^{\prime }\left(t_{r},\nu \right)$ have similar amplitudes. Because the amplitude of ${\hat{I}}_{n}^{\prime }\left(t_{a},\nu \right)$ varies across a data set in some cases, the definition of quality was expanded to [31]

(5) $$\begin{array}{c} Q_{n}\left(t_{a},t_{r}\right)=\frac{\max\left\{{\hat{I}}_{n}^{\prime }\left(t_{a},\nu \right)⊗{\hat{I}}_{n}^{\prime }\left(t_{r},\nu \right)\right\}}{∥{\hat{I}}_{n}^{\prime }\left(t_{a},\nu \right)∥\cdot ∥{\hat{I}}_{n}^{\prime }\left(t_{r},\nu \right)∥}. \end{array}$$

Equations (4) and (5) are identical for two measurements with the same amplitude. However, the use of a geometric mean of the auto-correlations for ${\hat{I}}_{n}^{\prime }\left(t_{r},\nu \right)$ and ${\hat{I}}_{n}^{\prime }\left(t_{a},\nu \right)$ is more appropriate when normalizing the cross-correlation maximum when the amplitude of ${\hat{I}}_{n}^{\prime }\left(t_{a},\nu \right)$ changes significantly.

### 2.3. Spectral Shift Reconstructions

To dynamically vary the reference when processing a set of OBR measurements, the best method must be determined to reconstruct the spectral shifts as a function of time and position. Contributors to this project were able to resolve larger spectral shifts beyond the capabilities of a static reference-based technique [31], and further optimization of spectral shift quality was hypothesized. The most systematic approach would be to calculate incremental spectral shifts for all positions relative to all previous measurements. Organizing the data in this manner produces a graph for each spatial position, with time serving as a nodal index, and the degrees of correlation (qualities) between each target-reference scan pair serving as the weights of the edges connected the nodes. Using this graphical framework, large OBR data sets can be analyzed using an analog of digital signal processing for data indexed in graphs [36,37]. In this work, OBR data sets were graphically analyzed, and optimal reconstructions of temperature-induced spectral shifts were developed using quality as the figure of merit that was optimized. Two different data sets were analyzed—one from an optical fiber with inscribed femtosecond FBGs, and one from a standard singlemode optical fiber without gratings—to evaluate the effect of SNR on the optimized reconstructions. This is the first analysis that optimizes spectral shift reconstructions of OBR data based on quality to ensure that the spectral shifts are calculated by cross-correlating spectra with the highest degree of correlation that is theoretically possible. Furthermore, this sensor-by-sensor optimization allows for the possibility that different portions of the FUT require different reference measurements in order to optimize the quality of the measurement as a whole.

## 3. Materials and Methods

### 3.1. Experimental Setup

The non-grated fiber tests were performed with an ordinary singlemode optical fiber (SMF-28; Corning Incorporated, Corning, NY, USA) that was terminated with a 10 cm coreless termination fiber (FG125LA; ThorLabs, Newton, NJ, USA) and freely suspended in a furnace (HF TAP 1210B; Evenheat Kiln, Inc., Caseville, MI, USA) during heating to temperatures of 100, 350, 550, 750, and 950 °C. The temperature was held for 40 min at each step and monitored using a Type K thermocouple. The grated fiber test was performed using a pure silica core, F-doped silica cladding singlemode optical fiber inscribed with 12 femtosecond Type-II FBGs (FemtoFiberTec, Goslar, Germany) centered near 1550 nm with a reflectivity less than 10%. This fiber was freely suspended in the same furnace and heated to 1130 °C with steps programmed to reach 350, 550, 750, 950, and 1150 °C, each held for 40 min.

### 3.2. Network Formulation of OBR Data

As described in Section 2.2, ${\hat{I}}_{n}^{\prime }\left(t_{a},\nu \right)$ is determined at times t∈T and sensor positions $x_{n}\forall n\in N$. An undirected graph $G_{n}=\left(T,A_{n}\right)$ can be formed by considering the time series measurements for sensor *n* at times *T* as the nodes and the spectral shift qualities as the weights of the edges connecting the nodes. The term $A_{n}$ is the adjacency matrix defined as

(6) $$A_{n}=\left[\begin{array}{ccccc} 0 & Q_{n}\left(t_{1},t_{0}\right) & \cdots & Q_{n}\left(t_{T-1},t_{0}\right) & Q_{n}\left(t_{T},t_{0}\right)\\ 0 & 0 & \cdots & Q_{n}\left(t_{T-1},t_{1}\right) & Q_{n}\left(t_{T},t_{1}\right)\\ \vdots & \vdots & \ddots & \vdots & \vdots \\ 0 & 0 & \cdots & 0 & Q_{n}\left(t_{T},t_{T-1}\right)\\ 0 & 0 & \cdots & 0 & 0 \end{array}\right],$$

with non-zero terms above the main diagonal. It should be noted that $G_{n}$ is functionally considered directed under considerations of causality, meaning the target scan index must be larger than the index of the reference scan.

### 3.3. Software Implementation and Data Usage

**Figure 3:**
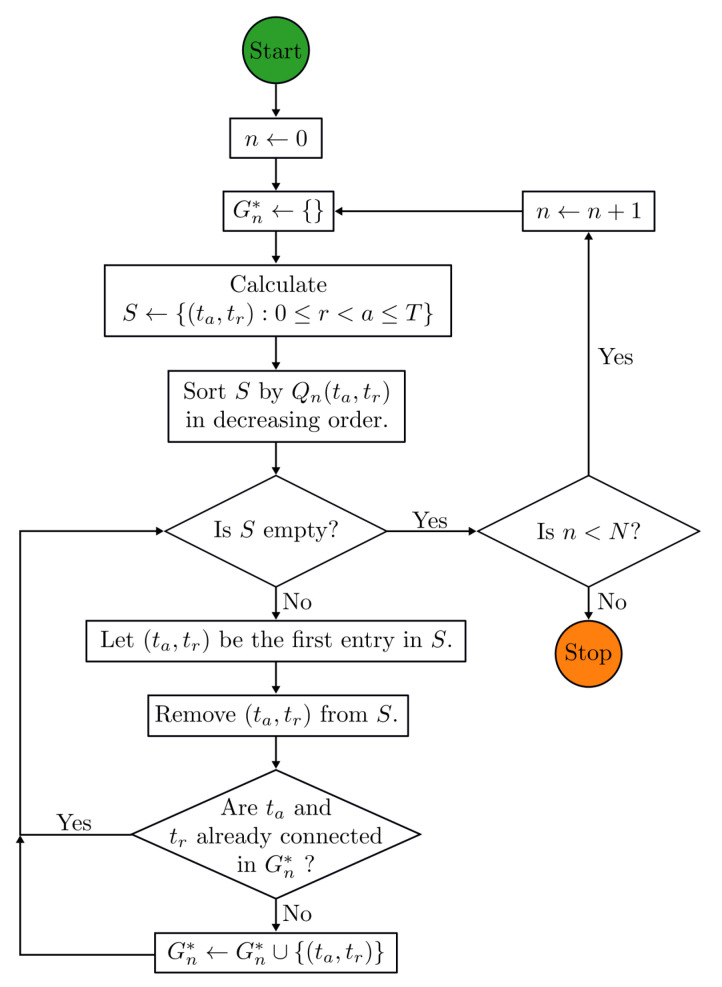
Flow diagram for Kruskal’s algorithm, where $G_{n}^{*}$ indicates a maximum spanning tree of $G_{n}$.

Optimal spanning trees are a classical structure in computer science consisting of an optimally selected, cycle-free subset of edges (in the present case weighted by the quality metric *Q*) that span all time-indexed nodes *t* for sensor *n* comprising $G_{n}$. Kruskal’s algorithm [38] and Prim’s/Jarník’s algorithm [39,40] take the list of quality-weighted edges connecting the nodes *t* and generate an optimal spanning tree. Kruskal’s algorithm, which was selected for the present work, begins by sorting all edges in order of descending quality. Then, a resulting set of edges is created incrementally by iterating across this list: if an edge connects a pair of nodes not mutually reachable in the existing result set, then the new edge is added to the result set. Otherwise, the edge is passed over. Kruskal’s algorithm is greedy, in the sense that it relies on optimizing connections between all nodes to produce a global solution (Figure 3). The final step is to re-index—or root—the spanning tree in order of time stamps so that all child nodes *t* can be connected back to $t_{0}$ through each immediately preceding node, referred to as the *parent*. To calculate cumulative spectral shifts Δν between a target node *a* and a reference node *r*, the spectral shift is the cumulative sum of the spectral shift between *a* and *r*.

A secondary goal of this work was to develop software tools to provide greater flexibility in the analysis methodology and to obtain a speed increase over that of the post-processing tools provided by Luna Innovations. Previous studies have utilized TCP/IP [31], human input emulation [3] to interact with commercial analysis software, or they have relied on analysis-specific code [22,24,28,29]. To build a more robust toolset for analyzing OBR data which could be easily integrated with other tools, the algorithms and supporting code used in the present work were developed around standard Python (v3.8.8) libraries Numpy (v1.19.2) and Scipy (v1.6.1). Least recently used (LRU) caching and threading were used to improve the speed of the analysis.

## 4. Results

### 4.1. Graphical Optimization of Non-Grated Fibers

**Figure 4:**
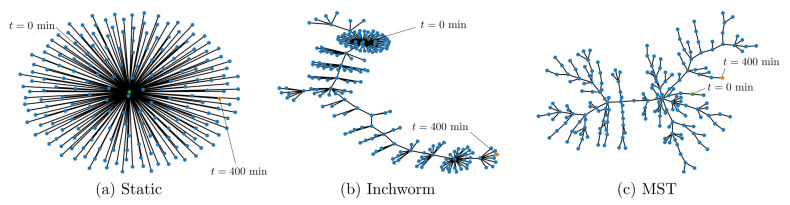
Examples of graphs generated from a heated SMF-28 optical fiber using a static reference (**a**), the inchworm algorithm [31] (**b**), and a maximum spanning tree (MST) algorithm (**c**). The node representing the initial measurement (t=0 min) is shown in yellow, and the final measurement (t=400 min) is shown in green.

Graphs $G_{n}=\left(T,A_{n}\right)$ were generated using a static reference, an adaptive reference approach using the inchworm algorithm [31], and then calculating the the quality between every possible pair of timestamp indices prior to performing a graphical optimization (MST). Graphical representations of the static reference method and the inchworm algorithm were generated for comparison, along with the graph generated as part of the graphical optimization scheme which utilizes Kruskal’s algorithm to generate a time-indexed MST. Figure 4 shows these graphical representations for the data obtained from an SMF-28 optical fiber heated to 950 °C and an optical fiber inscribed with femtosecond Type-II FBGs heated beyond 1100 °C. These data sets were used because the static reference method produced spurious spectral shift reconstructions, so it was postulated that a more sophisticated data processing scheme might resolve a larger portion of the data. The static reference method produced star graphs as shown in Figure 4a, with all nodes represented as “leaves” of the node representing the initial measurement at $t_{0}$. The inchworm algorithm produced graphs with few “non-leaf” vertices, similar to the caterpillar trees shown in Figure 4b, whereas the MST algorithm produced the most branching graphs, as shown in Figure 4c.

**Figure 5:**
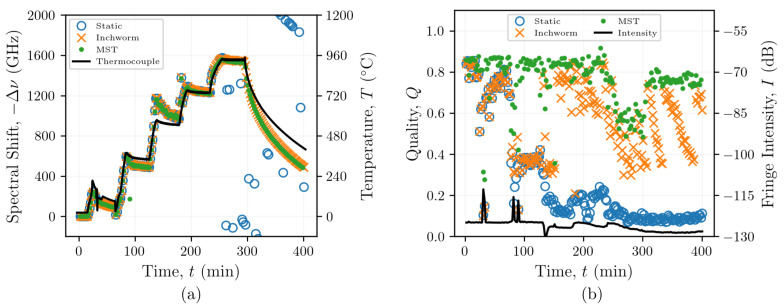
Spectral shifts (**a**) and qualities (**b**) of reconstructions of data from a heated SMF-28 optical fiber using the static reference, inchworm algorithm, and MST post-processing methods.

The branching structures of the graphs illustrate how each reconstruction method can adapt to elevated temperatures which alter the Rayleigh backscatter profile for Δν≤−1200 GHz (note that all spectral shifts are negative), which appeared to fluctuate rapidly in time, as shown in Figure 5a. As in previous findings [31], the static reference method failed to produce meaningful spectral shifts at temperatures in excess of 700 °C, whereas the inchworm and MST methods yielded similar spectral shift reconstructions without significant fluctuations for temperatures up to 950 °C. Both the inchworm and MST methods were capable of resolving spectral shifts Δν≤−1500 GHz, as shown in Figure 5a.

The quality *Q* provides insight into the mechanisms underlying the spurious spectral shift reconstructions produced by the static reference method. The initial quality is >0.8, but it decreases to a level between 0.6 and 0.7 up to 80 min. At 80 min, *Q* decreases to 0.4 concomitant with a temperature increase from 150 to 300 °C, as illustrated in Figure 5b. Despite these lower qualities, the spectral shift reconstruction remained consistent with those obtained using the inchworm and MST methods, which maintained much higher qualities. At 250 min (950 °C), the decrease in quality occurred simultaneously with a slight decrease in the Rayleigh backscatter intensity. The changes in the Rayleigh backscatter profile suggest structural changes in the FUT which would affect the ability of the algorithm to identify the true cross-correlation peak used to calculate spectral shift, as shown in Equation (2). This is also evidenced by the low quality at 400 min compared to 120 min when the fiber at the same temperature (Figure 5b). Instead, false peaks in the cross-correlation spectrum lead to the spurious fluctuations which are generated using the static reference method beyond 250 min. These data suggest that the static reference method relies on increasingly poor correlations as the absolute spectral shift increases, which introduces significant errors, resulting in the identification of false peaks in the cross-correlation analysis, as indicated by the decreasing quality. The ability of the inchworm and MST methods to adapt as the cross-correlation peak decreases solves this problem and generally results in qualities >0.25 throughout the experiment. The inchworm and MST methods enabled spectral shift reconstructions even while the Rayleigh backscatter intensity decreased, with the MST generally yielding the highest qualities of the three methods, as intended.

### 4.2. Graphical Optimization of Fibers with Type-II FBGs

**Figure 6:**
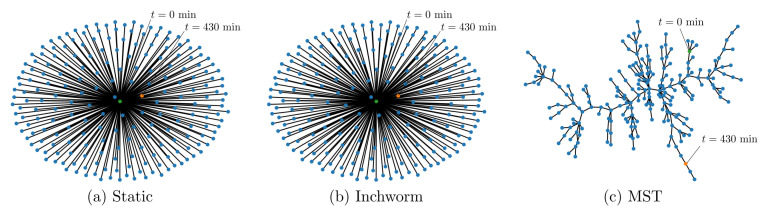
Examples of graphs generated from optical fibers with Type-II FBGs using a static reference (**a**), the inchworm algorithm [31] (**b**), and a maximum spanning tree (MST) algorithm (**c**). The node representing the initial measurement (t=0 min) is shown in yellow, and the final measurement (t=430 min) is shown in green.

FBGs are inscribed in optical fibers to create stronger reflections than those in non-grated fibers. This increases the SNR and therefore improves the ability of the post-processing algorithm to identify true cross-correlation peaks. The FBG data set analyzed in this work involved heating an optical fiber with 12 Type-II FBGs to temperatures up to 1130 °C. As with the non-grated fiber, graphs were produced after analyzing these data using the static reference, inchworm, and MST algorithms (Figure 6). Both the static reference and inchworm algorithms generally produced graphs without branching (radii close to unity), as seen in Figure 6a,b, whereas the MST algorithm consistently produced larger radius graphs with more branching, as shown in Figure 6c. Interestingly, the graph branching does not appear to be correlated with the magnitude of the spectral shift in the same manner that was observed with the non-grated FUT. Each of the spectral shift reconstructions of data obtained from the fiber with Type-II FBGs appeared to produce consistent results, regardless of the reconstruction method, up to 300 min. This corresponds to a spectral shift of approximately −2000 GHz at 1130 °C. Therefore, it appears that the benefit of the inchworm and MST methods may be limited for optical fibers with Type-II FBGs.

**Figure 7:**
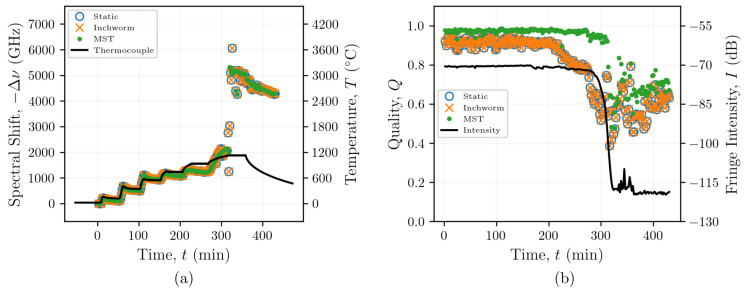
Spectral shifts (**a**) and qualities (**b**) from reconstructions of data from a heated optical fiber with Type-II FBGs using the static reference, inchworm algorithm, and MST post-processing methods.

Even though the same cross-correlation peaks were identified using the static reference, inchworm, and MST algorithms, the large increases in spectral shift beyond 300 min indicate that the peaks identified in the analysis did not correspond to physical changes in temperature. The origins of these seemingly non-physical changes in spectral shift beyond 300 min could be attributed to annealing of the Type-II FBGs. Figure 2c shows how the reflected light intensities from the FBGs decreased from ∼50 dB at the start of the test to ∼130 dB. This decrease is similar to those of the non-grated fiber shown in Figure 7a near 400 min, when the temperature was 1130 °C. Figure 2d shows multiple peaks appearing in the reflected spectrum from one sensing location after heating to 1130 °C. It is likely that there are contributions to this spectrum other than those from the annealed grating that could be causing the non-physical changes in spectral shift that were observed following the reconstructions.

### 4.3. Resolution of Large Spectral Shifts

**Figure 8:**
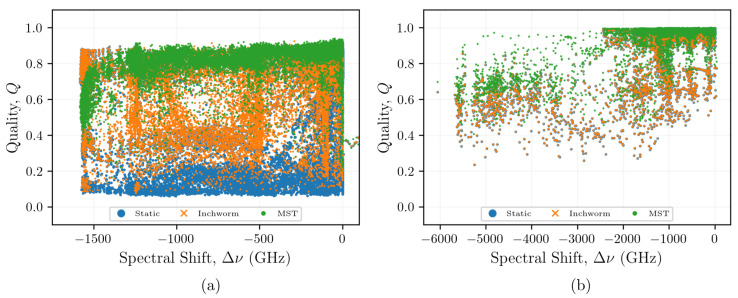
Quality determined using each reconstruction method compared to the spectral shift magnitude determined using the MST algorithm for sensors within the non-grated SMF-28 optical fiber (**a**) and the fiber with Type-II FBGs (**b**).

In all OFDR-based measurements, changes in temperature or strain do not easily resolve when the SNR is low and a large stimulus (temperature or strain) is applied. This is particularly true when using non-grated fiber because of the relatively weak signal from the Rayleigh backscatter. In this work, when a static reference was used, there appeared to be a threshold spectral shift beyond which the algorithm could no longer reliably determine the spectral shift. This inability to reliably determine the spectral shift coincided with a significant reduction in quality. The relationship between spectral shift and quality was characterized by plotting qualities from the SMF-28 fiber (Figure 8a) and the FUT with Type-II FBGs (Figure 8b), obtained using each of the reconstruction algorithms, against the spectral shifts reconstructed by the MST algorithm for each sensor at each time. For the SMF-28 fiber, the quality for poorly correlated measurements with larger spectral shift magnitudes appears to range from 0.1 to 0.15. To maintain a sufficiently high quality (≥0.15) and to mitigate the effects of false peak detection with the cross-correlation operation, the absolute magnitude of the spectral shift should not exceed ∼600 GHz when using a static reference (Figure 5b). The spectral shift quality is maintained at Q≥0.15 for the inchworm algorithm and Q≥0.2 for the MST algorithm for spectral shifts up to −1500 GHz, although the majority of the quality data exist far above these lower bounds. As expected, these data indicate that maintaining a high quality enables resolution of larger spectral shifts.

The spectral shifts reconstructed from the FUT with Type-II FBGs generally yielded higher qualities than those obtained with the SMF-28 reconstructions, with Q≥0.3 for the static reference, inchworm, and MST algorithms. The ability to maintain a higher quality over a similar range of spectral shifts is attributed to the higher SNRs that are achieved using FBGs compared to non-grated SMF-28 fibers. This suggests that adaptive reconstruction methods may be unnecessary to reconstruct distributed spectral shift measurements from FUTs with Type-II FBGs, or other fibers with enhanced Rayleigh backscatter intensities. However, some applications may require adaptive reconstruction methods, even when using FBGs. These applications are discussed in Section 5.

### 4.4. Computational Efficiency

**Figure 9:**
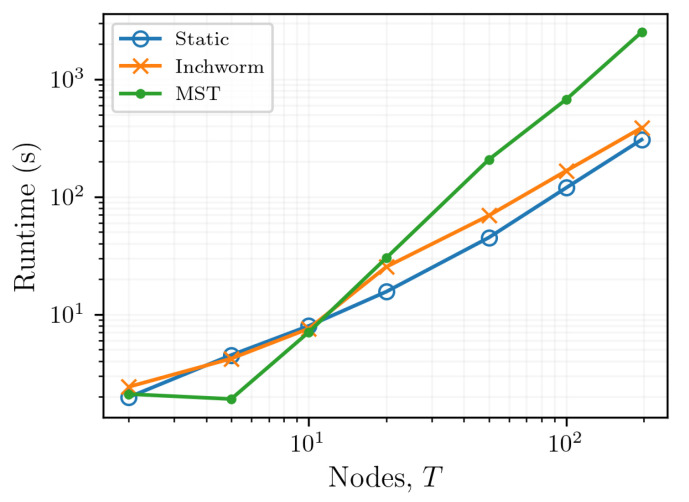
The computational efficiency of the static reference, inchworm, and MST reconstruction algorithms.

The computational effort required to reconstruct spectral shifts from a set of OFDR data is particularly important when analyzing large data sets or when considering integrating these processing schemes into the data acquisition process. In the present context, the computational effort required to analyze OBR data using a static reference is O(NT), in big-O notation (Figure 9). This is the simplest case because only a single reference-target pair must be calculated for each time index and each distributed sensor. In contrast, local optimization of the quality between target-reference pairs inherently requires comparison of a single target data set with multiple reference data sets. The MST algorithm accordingly requires calculating the quality between each pair of scans, which has an efficiency of $O(NT^{2})$. The inchworm algorithm, as published by Sweeney et al. [31], has an efficiency bounded from above by $O(NT^{2})$ and from below by Ω(NT), depending on the lowest quality value calculated between a target-reference data pair.

## 5. Discussion

### 5.1. Comparison to Previous Works

Distributed and quasi-distributed sensing techniques based on fiber optic sensors are attractive for a wide range of applications and are increasingly being considered for extreme environments because fibers offer immunity to electromagnetic interference, they provide resilience to conditions (e.g., temperature, ionizing radiation, chemical exposure) which damage semiconductors, and they offer the possibility of remote operation. For example, optical fiber-based temperature sensors have been proposed for measuring core temperature distributions in nuclear reactors during operations based on their ability to survive temperatures beyond 1000 °C [41] and fast neutron fluences of >10${}^{20}$ n/cm^2^ [42]. Despite the fact that the fibers themselves can survive these conditions, previous work has shown that spectral shift reconstructions using a static reference are unable to reliably determine spectral shifts at temperatures greater than 600 to 700 °C and under neutron irradiation [21,43]. However, if the fiber degradation processes resulting from exposure to high temperatures and/or neutron irradiation occur slowly enough, post-processing techniques utilizing an adaptive reference were able to resolve spectral shifts in these harsh environments [31].

The inchworm algorithm does not allow for sensor-by-sensor optimization and instead varies the reference only when the minimum quality calculated for all sensors (i.e., spatial locations) drops below a threshold value. Eliminating sensor-by-sensor optimizations reduces the computational expense but may prevent the algorithm from resolving spectral shifts at all locations, even when only a few sensors cannot be resolved. The graphical approaches explored in this work are based on algorithms developed to produce optimal spanning trees. This enables the optimal reconstructions of OBR data by maximizing the quality calculated between each target-reference pair. Furthermore, this optimization is independently performed for each sensor, instead of the minimum quality being evaluated across all spatial positions.

**Figure A1:**
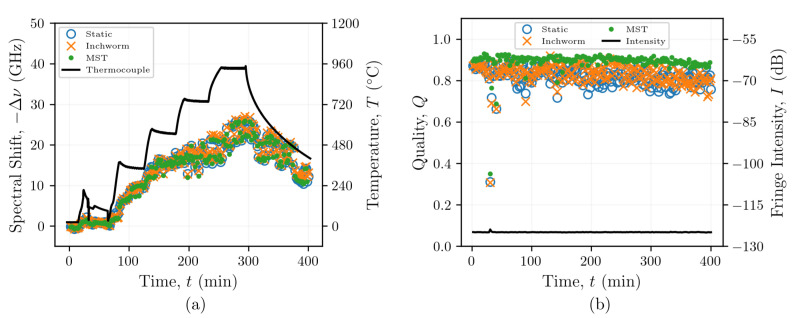
Spectral shifts (**a**) and qualities (**b**) of reconstructions of data from the region of the SMF-28 optical fiber outside of the furnace produced using the static reference, the inchworm algorithm, and MST post-processing methods. The temperatures indicated by the black line are indicative of the furnace temperature at the time of recording and are the cause for the smaller spectral shifts, relative to Figure 5.

**Figure A2:**
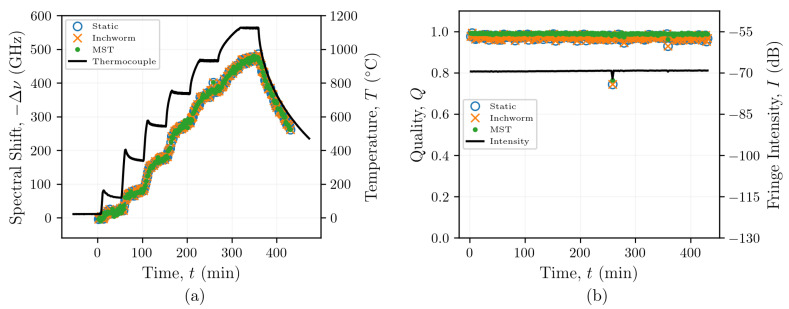
Spectral shifts (**a**) and qualities (**b**) of reconstructions of data from the region of the FBG fiber optical fiber outside of the furnace produced using the static reference, the inchworm algorithm, and MST post-processing methods. The temperatures indicated by the black line are indicative of the furnace temperature at the time of recording and are the cause for the smaller spectral shifts, relative to Figure 7.

While the focus of the present work is on adaptive methods to resolve large spectral shifts, the inchworm and MST post-processing methods produce reconstructions for smaller-magnitude spectral shifts which are almost identical to those produced by the static method. To illustrate this, data from positions along the SMF-28 and FBG fibers located outside of, but still near, the furnace were reconstructed using each of the three reconstruction methods (Appendix A, Figure A1 and Figure A2). For both fibers, the spectral shift reconstructions produced by the MST and inchworm methods were in excellent agreement with the static reference method and well within the bounds suggested by Wood et al. [21] (Appendix A, Figure A1a and Figure A2a). The quality of these measurements also remained high throughout the entire reconstruction, with the SMF-28 data remaining above 0.75 (Appendix A, Figure A1b) and the FBG data remaining above 0.90 (Appendix A, Figure A2b), excluding outliers. As expected, the MST yielded reconstructions with the highest quality for both the SMF-28 and FBG fibers, consistent with the data from the fiber positions inside the furnace (Figure 5b and Figure 7b), because the algorithm was designed to maximize quality.

The MST algorithm developed in the present work maximizes the quality for all sensor positions and all times by adaptively varying the reference as a function of time. This allows the algorithm to determine incremental changes in temperature or strain over time, even if the spectral features of the backscattered light change. However, large spatial variations in temperature or strain along the length of an optical fiber can still present challenges, particularly if these variations occur over lengths comparable to the sensor gauge length. This issue is not specific to the MST algorithm and is a known issue for all OFDR-based distributed sensing techniques. Although varying the reference will not help resolve large spatial variations in temperature or strain, several other techniques have been proposed to this end. For example, summing the upstream strains and shifting the gauge position downstream by the cumulative calculated upstream strain has been demonstrated to improve OFDR measurements [29,44]. The gauge length could also be decreased to reduce the variation in temperature or strain over the gauge at the expense of temperature or strain resolution [45]. These techniques could be used in combination with the adaptive methods presented herein to resolve both temporal and spatial variations in temperature or strain. This implementation could be considered in future work.

### 5.2. Computational Efficiency

If the cost of a multi-edge path through a graph is assumed to be the product of the costs of its constituent edges, or the sum or root-square-sum of any other monotone aggregating operation (ensuring that the cost of two edges in series is at least as expensive as the cost of either edge individually), then a form of Dijkstra’s shortest path algorithm can be used to optimize the path length to each node with this new measure of path quality. The networks that result from maximizing the product of qualities along each path to the root resulted in graphs similar to the static reference, indicating that cumulative product quality is not the appropriate optimization target to improve resolution of the spectral shift reconstruction. In contrast, when the cost of a path is measured by the maximum cost (minimum quality) of its component edges, Dijkstra’s algorithm computes an optimal spanning tree. Kruskal’s algorithm, which also produces the optimal spanning tree, selects the path from the root to a given node that optimizes the worst edge along that path.

Kruskal’s algorithm extends the graph radius, as well as the capacity for the reconstruction, to resolve larger spectral shifts and to retain a higher SNR than that allowed by a static reference scheme. Therefore, the optimal reconstruction produced in this way is the best that can be produced given a particular data set. However, the guarantee of a maximum quality reconstruction comes at the cost of efficiency; because the MST requires the computation of every target-reference pair for each sensor, it has an efficiency of $O(NT^{2})$. Generating an MST requires greater computational resources than those required by methods based on a static reference (O(NT)). To overcome this decreased efficiency, the MST-based scheme presented in this work was implemented using threads to parallelize the calculation of the correlation coefficient by doing so for multiple sensors at the same time, such that each of the *n* sensors had an effective efficiency of $O(T^{2})$. This parallelization is scalable and could be implemented on a cluster to improve the post-processing runtime. This parallelization is particularly useful for long FUTs where N≫T, but it only provides marginal performance improvements when T≪N. The inchworm algorithm [31], which places a sufficiency condition on the correlation coefficient between two scans, exists between the efficiencies of the MST and static reference methods. The efficiency of the inchworm algorithm is Ω(NT) when the first scan $t_{0}$ is sufficiently highly correlated to each consecutive scan, and $O(NT^{2})$ when the only poor correlations exist between each pair of scans.

### 5.3. FBG Applications

Figure 5 shows how the inchworm and MST algorithms significantly improved the qualities for the SMF-28 fibers without FBGs, particularly at higher temperatures. Processing the data from the optical fiber Type-II FBGs using the inchworm and MST algorithms did not show the same improvements in quality, as indicated in Figure 7b. This suggests that FBGs do not require the use of adaptive algorithms to reconstruct their spectral shift history for temperatures beyond 1000 °C (Figure 8). This is not surprising, as the gratings inscribed in these fibers are designed to increase the amplitude of the backscattered light at each grating by locally altering the refractive index of the fiber, resulting in reflections that are orders of magnitude larger than the Rayleigh backscattered light from random density fluctuations, as seen in Figure 2c,d. However, once the gratings start to anneal at temperatures beyond 1000 °C, as evidenced by the reduction in fringe intensity shown in Figure 7b, the qualities still remained relatively high (>0.4), regardless of the reconstruction method. Therefore, if quality were the only metric for determining whether the measurement is reliable, then the non-physical increases in spectral shift at around 300 min could be incorrectly interpreted as a rapid increase in temperature.

The high quality that was observed even after the gratings were annealed suggests that there may be contributions to the optical spectra at these locations besides those from the original gratings. For example, Figure 2d shows how multiple peaks appear in the reflected spectrum from one sensing location after heating to 1130 °C. These peaks could cause the cross-correlation to shift the reference spectrum, which contains the original grating peak, to align with one or more of the artificial peaks, resulting in a non-physical spectral shift with a high quality due to the similarity in peak profiles. The source of the artificial peaks that can be seen after the gratings are annealed could be related to ghost gratings and/or spectral shadowing [46,47]. In the present work, some of the original gratings located outside of the high-temperature region were not annealed when the high-temperature region was heated to 1130 °C, as shown in Figure 2c. Multiple reflections from these high reflectivity gratings could increase the optical path length; this may explain why reflections from these gratings could be visible at locations that correspond to gratings located further downstream along the fiber within the high-temperature region. Once the FBGs are annealed, the artificial peaks could become the prominent features in the optical spectra, causing the cross-correlation operation to shift the reference spectra to align with these artificial peaks. This would explain the sudden change in spectral shift seen in Figure 7a near 300 min.

These results suggest that the adaptive post-processing methods relying on quality to guide the reference selection may not be necessary for resolving data taken from fibers with FBGs because they do not provide more spectral shift fidelity than the more efficient static reference method (see Figure 5 and Figure 7). Because these adaptive reference algorithms have been shown to resolve spectral shifts in non-grating fibers at temperatures up to 1000 °C, it is still possible that these techniques could resolve spectral shifts in fibers with FBGs, even after the gratings are annealed. However, this work shows that challenges related to ghost gratings or other effects from non-annealed gratings along the same fiber could impede resolution of spectral shifts after some of the gratings are annealed. Therefore, for applications that could expose FBGs to temperatures beyond the annealing point of the gratings, adaptive reference methods could still be used to resolve spectral shifts after the gratings are annealed if the FBGs do not extend outside the high temperature region.

## 6. Conclusions

Optical backscatter reflectometry relies on reflections from scattering centers within a singlemode optical fiber to measure changes in temperature and mechanical strain. The cross-correlation coefficient between a target and a reference measurement is used as a metric to characterize the quality of the relationship between the two measurements. This work provides a theoretical framework for the quality metric and has demonstrated the implementation of this framework to optimize the achievable spectral quality for a given data set. This framework was applied to two sets of OFDR data from optical fibers with and without femtosecond FBGs heated to temperatures up to or exceeding 1000 °C. Results showed that the MST algorithm was able to reconstruct spectral shifts from non-grated fibers for all tested temperatures; this is similar to what was achieved using the inchworm algorithm. For fibers with inscribed FBGs, the MST and inchworm algorithms did not provide any additional benefit due to the higher SNR of the FBGs, but it remains possible that these algorithms could resolve spectral shifts from FBGs, even after the FBGs are annealed. For these cases, it is recommended that all FBGs be exposed to high temperatures so that the gratings that do not anneal do not impact measurements in the high-temperature region, as observed in the present work. The MST is theoretically capable of resolve spectral shifts at some locations, even when an acceptable correlation cannot be obtained at other locations. This increased robustness comes at the cost of increased computation time during processing. However, because the MST operates on each sensor individually, the processing can be parallelized to greatly improve data processing times.

## Abbreviations

The following abbreviations are used in this manuscript:

FBG	Fiber Bragg grating
MST	Maximum spanning tree
OBR	Optical backscatter reflectometry
OFDR	Optical frequency domain reflectometry
SNR	Signal-to-noise ratio
